# Role of Memory T Cells and Perspectives for Intervention in Organ Transplantation

**DOI:** 10.3389/fimmu.2015.00473

**Published:** 2015-09-14

**Authors:** Kailin Lin, Song Chen, Gang Chen

**Affiliations:** ^1^Institute of Organ Transplantation, Tongji Hospital, Huazhong University of Science and Technology, Wuhan, China; ^2^Key Laboratory of Organ Transplantation, Ministry of Education, Wuhan, China; ^3^Key Laboratory of Organ Transplantation, Ministry of Public Health, Wuhan, China

**Keywords:** memory T cells, transplantation, allograft rejection, tolerance, therapy

## Abstract

Memory T cells are necessary for protective immunity against invading pathogens, especially under conditions of immunosuppression. However, their presence also threatens transplant survival, making transplantation a great challenge. Significant progress has been achieved in recent years in advancing our understanding of the role that memory T cells play in transplantation. This review focuses on the latest advances in our understanding of the involvement of memory T cells in graft rejection and transplant tolerance and discusses potential strategies for targeting memory T cells in order to minimize allograft rejection and optimize clinical outcomes.

## Introduction

Adaptive immune responses depend on the ability to recognize and eliminate recurrent pathogens, resulting in the generation of memory lymphocytes. The capacity of memory T cells to rapidly mobilize and initiate a potent recall response enhances protective immunity against previously encountered pathogens. However, the characteristics of memory T cells, with their lowered activation thresholds and lower susceptibility to conventional immunosuppressive agents, also makes them a significant obstacle to successful transplantation (1).

In the context of transplantation, three independent mechanisms for the generation of alloreactive memory T cells have been described. First, since memory T cells can be generated directly during primary immune responses, alloantigenic stimulation of naïve T cells serves as the most direct source of alloreactive memory T cells (2). Second, alloreactive memory T cells can also be generated through homeostatic proliferation, a spontaneous process that occurs in response to transient lymphopenia and induces the proliferation and differentiation of naïve T cells into memory cells; in transplantation, severe lymphopenia is often seen after T-cell depletion therapy (3). Third, memory T cells generated in response to infectious or environmental antigens have the potential to cross-react with donor allogeneic MHC molecules (4), creating a situation known as heterologous immunity. In recent years, a broader perspective has been established with regard to our understanding of the basic biology of memory T cells. Also, new approaches for modulating memory T cells have been designed and tested in various models, including transplantation. In this review, we mainly discuss the role of memory T cells in transplantation and explore the development of therapeutic strategies that can directly target memory T cells.

## The Role of Memory T Cells in Transplantation

In contrast to naïve T cells, memory T cells have lower activation thresholds and are less dependent on costimulation signals (5), are more resistant to regulation by regulatory T cells (Treg) (6), and are less susceptible to conventional immunosuppressive agents (1). These features make memory T cells a significant obstacle to successful transplantation. However, the exact mechanisms involved remain unclear. In the pursuit of innovative and effective strategies to inhibit or deplete memory T cells and improve the clinical outcomes, a profound and comprehensive understanding of its role in transplantation is urgently required. Therefore, studies based upon animal models and clinical observations have therefore been established to directly or indirectly demonstrate the impact of memory T cells on transplantation and to elucidate the relevant mechanisms.

### One chief culprit for rejection

In addition to their role in protective immunity, memory T cells also take part in mediating transplant rejection (7). It is well understood that donor-specific memory T cells mediate the so-called second-set rejection that is rather difficult to block or inhibit (8, 9). In animal models, memory T cells alone are sufficient to trigger rejection, during which they are among the first cell types infiltrating the grafts (10). In recent observations from clinical practice, the expansion of memory CD8^+^ T cells and memory CD4^+^ T cells has been found to be associated with acute cellular and antibody-mediated rejection, respectively, in liver transplantation (11, 12), and these findings are consistent with reported experimental outcomes.

In the absence of direct stimulation by alloantigen, memory T cells generated through either homeostatic proliferation or heterologous immunity seem to have the same ability to mediate transplant rejection. It has been demonstrated that the memory T cells generated via homeostatic proliferation act as potent effector cells in the rejection of heart and skin allografts (5). Likewise, heterologous immunity may also lead to the generation of alloreactive memory T cells (13). Functional heterogeneity of the human T-cell response induced by pathogens and vaccines has recently been described (14). Infection of B6 mice with parasitic or viral antigens, for instance, has been found to cause the generation of alloreactive memory T cells. As a result, heart grafts transplanted into these pathogen-recognizing mice demonstrate an accelerated rejection (15). It has also been reported that naïve untreated adult laboratory mice possess a repertoire of endogenous memory T cells that are naturally generated by environmental exposure (16). Endogenous memory T cells are not previously primed to donor antigens; however, a proportion of the endogenous memory T-cell repertoire in naïve mice is reactive with donor class I MHC molecules, and these T cells are rapidly detected and infiltrate into cardiac allografts within hours of reperfusion (17). In other selected models, memory T cells have been shown to be pivotal mediators of chronic allograft rejection, implying that memory T cells and chronic rejection may also be tightly associated (18, 19). Therefore, despite the different mechanisms by which they are generated, memory T cells are capable of mediating rejection.

The cognate interactions between memory helper T cells and B cells are also important for the development of allograft rejection. On the one hand, it has been reported that donor-reactive memory CD4^+^ T cells can provide CD40-independent help to B cells and induce high levels of alloantibodies that contribute to heart allograft rejection in mice (20). The CD40-independent help delivered by memory CD4^+^ T cells in alloreactive immune responses is mainly mediated by B cell activating factor (BAFF) and a proliferation-inducing ligand (APRIL), as targeting the BAFF cytokine network has been reported to be able to inhibit both humoral and cellular immune responses induced by memory CD4^+^ T cells (21). Additionally, IFN-γ secreted by pre-existing memory helper cells has been found to be required for the CD40-independent alloantibody responses, which determines both isotype and specificity of donor-reactive alloantibodies and can thus affect allograft (22). On the other hand, B cells can also provide help for T cells via multiple mechanisms promoting T-cell proliferation, differentiation, and survival to generate memory T cells. Experimental evidence has shown that activated T cells cotransferred with B cells give rise to more memory T cells than those transferred without B cells and upon recall, mediated accelerated rejection of skin allografts in mice (23). Therefore, inhibiting memory T/B cells interactions could possibly prevent not only alloantibody production but also generation of long-lived memory T cells, which may improve allograft survival.

### Turnoff: Tolerance-inducing or not?

Another important concern is the correlation between memory T cells and tolerance induction in transplantation. Memory T cells have been found to be highly resistant to tolerance induction, and tolerizing therapies that are effective in inducing allograft tolerance in naïve animals often fail to do so in memory-rich animals (24). A higher frequency of memory T cells in recipients is associated with a worse transplant outcome and poorer tolerance induction under conventional immunosuppression (25).

Memory T cells are distinguished by their reduced requirement for both TCR stimulation and costimulatory signals for recall responses. Showing differences in the expression of adhesion molecules (such as LFA-1, VLA-4, and CD44), cytokine receptors (such as CD122 and IL-15Rα), and apoptosis involving molecules of the Bcl-2 and caspase-3 families when compared to naïve T cells, donor-reactive memory T cells are relatively refractory to any blockade of conventional costimulatory pathways (26–29). In a seminal study, although blockade of costimulation by CD28 and CD154 effectively inhibited graft rejection in naïve recipients, animals that had previously been infected with viruses were found to be refractory to the tolerance-inducing effects of this costimulation blockade; while this study focused primarily on the CD8^+^ memory T-cell barrier, it is clear that both CD4^+^ and CD8^+^ donor-specific memory cells can constitute a barrier to costimulation blockade-induced tolerance (30). A study examining the efficacy of an approach combining costimulatory blockade and bone marrow or donor-specific transfusion (DST) to induce tolerance in non-human primate renal transplantation showed that higher pre-transplant precursor frequencies of donor-reactive memory T cells were correlated with a failure of tolerance induction and acute rejection of the grafts, whereas low pre-transplant frequencies of donor-reactive memory T cells predicted successful tolerance induction and long-term renal survival (31).

Memory T cells have also been shown to exhibit increased resistance to regulation by Treg. In a murine model, transferred CD4^+^CD25^+^ cells effectively inhibited the rejection mediated by naïve but not by memory CD4^+^ T cells (32). Similarly, Treg were found to be unable to regulate CD8^+^ alloreactive T-cell responses, a finding that was true for both naive Treg and alloantigen-primed Treg, suggesting that strategies to enhance the frequency and/or activation of alloantigen-specific Treg are unlikely to be effective against donor-reactive memory T cells (33). However, another report has suggested that the stimulation of human T cells *in vitro* with TLR-stimulated plasmacytoid dendritic cells results in the generation of CD8^+^FoxP3^+^LAG-3^+^CTLA-4^+^ Treg, which can inhibit alloreactive memory T-cell responses (34, 35). It has also been reported that human CD45RA^−^FoxP3^hi^ memory-type Treg use a different T-cell receptor (TCR) repertoire from conventional T cells and play an important role in controlling early immune activation (36). Thus, such regulatory effects might be of clinical importance in the pursuit of a desirable level of tolerance.

## Current and Potential Memory T Cell-Directed Intervention Therapies

If the negative impact of memory T cells on transplantation and their distinct hindrance of tolerance induction are taken into account, strategies to target memory T cells may offer a solution to improve transplant outcomes. The pursuit of memory T cell-directed therapies in transplantation has arisen from the observation that standard immunosuppressive agents often have undesirable effects on memory T cells. Nevertheless, studies in several experimental models have suggested that targeting the infiltration, proliferation, activation, and the intrinsic apoptosis pathway of memory T cells and the blocking of memory T/B cells interactions may be promising therapeutic approaches.

### Blocking the infiltration of memory T cells

After organ transplantation, the alloreactive memory T cells first infiltrate the grafts, then proliferate and mediate significant injury. Therefore, inhibition of their initial entry into the graft may improve allograft survival. There have been numerous attempts to control memory T-cell infiltration in transplant models. For example, the administration of FTY720, the sphingosine-1 phosphate receptor agonist, has been found to lead to a quarantine of donor-specific memory CD4^+^ T cells in the peripheral lymph nodes and to postpone heart allograft rejection in mice (37). In this situation, the administration of FTY720 prevents the migration of lymphocytes from thymus and peripheral lymphoid tissues, sequesters T cells in the lymph nodes, and inhibits them from infiltrating the grafts. However, this sequestration does not affect the ability of donor antigen-reactive CD4^+^ T cells to facilitate the helper signals needed to stimulate a donor-specific antibody response, which plays a vital role in the graft loss in these FTY720-treated recipients.

Other studies have also demonstrated that the disruption of adhesion molecules, e.g., leukocyte integrins such as LFA-1 and VLA-4, is effective in preventing the infiltration of memory T cells into grafts (38, 39). Anti-LFA-1 or anti-VLA-4 monoclonal antibodies can attenuate donor-reactive memory recall responses and reduce T-cell trafficking into allografts in mouse models, resulting in a prolongation of allograft survival (40). However, a preclinical test of the anti-LFA-1 agent Efalizumab in primate renal transplantation suggested that this agent evokes a higher rate of EBV-associated malignancy despite the promising outcomes produced for the grafts (40, 41). Taken together, these successful treatments in animal models, though not currently available in the clinic, suggest that targeting trafficking molecules on memory T cells may be a valid approach, but further investigation is urgently needed to provide a full evaluation of its clinical validity and potential side effects.

### Suppressing the proliferation of memory T cells

Another potential therapeutic strategy is targeting the proliferation of memory T cells induced by cytokine and TCR signaling. Janus kinase-3 (JAK-3), the downstream receptor of the common γ chain, binds a magnitude of cytokines, including IL-2, IL-7, IL-9, IL-15, and IL-21 (42). These cytokines have been shown to play pivotal roles in the generation, maintenance, and proliferation of memory T cells. Tofacitinib (CP-690550), a highly selective and potent JAK-3 inhibitor, has been shown to prevent allograft rejection in both rodent and non-human primate models and may offer a novel means of targeting memory T cells without TCR inhibition (43).

Cell proliferation and survival signaling may also be induced through the TCR pathway. Nuclear factor-κB (NF-κB), a protein complex that regulates DNA transcription, plays an important role in the TCR pathway (44). The NF-κB blocker 15-deoxyspergualin can block the activation of donor-specific memory CD8^+^ T cells and has been shown to induce skin allograft survival in a mouse model in combination with costimulatory blockade (45). The inhibition of NF-κB also suppresses the proliferation of rapamycin-resistant memory T cells in non-human primates (46).

### Inhibiting the activation of memory T cells

An increasing amount of evidence has shown that conventional blockade of costimulation has only a minimal effect on memory T cells. Several studies have suggested that memory T cells use alternative, unique costimulatory pathways for activation and effector activity. On CD8^+^ T cells, it has been shown that the engagement of 4-1BB (CD137) by its ligand provides both CD28-dependent and CD28-independent signals that lead to cytokine production, cell proliferation, augmented cytotoxic effector activity, and enhanced cell survival (47). In mouse models, blockade of the 4-1BB costimulatory pathway has been shown to be valid in prolonging the survival of intestinal, skin, and heart allografts (48, 49). Another costimulatory pathway that might account for the recall of memory T cells is the OX40/OX40L pathway (50). A deficiency in or blockade of OX40 has been shown to lead to an impairment of memory CD4^+^ T cell formation and to prolong the survival of heart and skin allografts in recipients. Anti-OX40L monoclonal antibody prolongs the secondary cardiac allograft survival on the basis of CD40/CD40L and LFA-1/ICAM-1 blockade, with the anti-OX40L mAb impairing the generation of memory T cells and up-regulating IL-10-producing Tregs, thereby inhibiting T-cell function (51).

### Depleting memory T cells

In addition to the strategies mentioned above, the most common therapeutic option for induction in transplantation is the administration of polyclonal antithymocyte globulin (ATG), which is produced in response to target thymocytes or T-cell lines (52, 53). Although ATG can effectively deplete alloreactive T cells, lymphopenia will induce compensatory proliferation that paradoxically supports memory T cells, favoring graft rejection and making it difficult to achieve or sustain operational tolerance of the graft (54, 55). However, several studies have shown that ATG may also play an important role in human Treg survival and expansion both *in vitro* and *in vivo* (56, 57). An increased ratio of Treg/T effector cells during ATG-induced homeostatic proliferation has been observed in rat kidney transplantation (58). Although memory T cells are much more resistant to depletion than are naïve T cells, Tregs proliferate to a significantly higher extent than do effector T cells, which suggests a biological preference of ATG for regulation rather than for promoting an effector immune function. In mice, it has been reported that pre-transplant administration of ATG results in increased efficacy in controlling donor-reactive memory T cells when compared to its peri-transplant administration (59). The application of ATG pre-transplantation results in a greater inhibition of pre-existing donor-reactive memory T-cell responses and a slower recovery of memory T cell counts than does peri-transplant treatment, making ATG-mediated depletion more efficient in prolonging allograft survival (59).

Some fundamental studies of the role of apoptosis in memory T cells have prompted us to target the apoptotic pathway to achieve functionally relevant depletion of memory T cells (58, 60). Regulation of the intrinsic apoptotic pathway by both pro- and antiapoptotic factors of the Bcl-2 family is critically important for the selection of T-cell clones for memory generation and for the maintenance of memory T cells. ABT-737, a small-molecule inhibitor of Bcl-2/Bcl-XL, efficiently induces apoptosis in alloreactive memory T cells *in vitro* and *in vivo* and prolongs skin graft survival in sensitized mouse recipients (26). Memory T-cell reduction produced by Bcl-2 inhibition seems to represent an important advance in the field of transplantation, which may benefit HLA-presensitized transplantation as well as tolerance induction.

### Blocking memory T/B cells interactions

As mentioned above, blocking interactions between memory T and B cells in transplantation may prevent not only alloantibody formation but also generation of long-lived memory T cells improving allograft survival. The short-term neutralization of BAFF alone or BAFF plus APRIL synergized with anti-CD154 monoclonal antibody was reported to prolong heart allograft survival in recipient mice containing donor-reactive memory CD4^+^ T cells, indicating that reagents neutralizing BAFF and APRIL might be used to enhance the efficacy of CD40/CD154 costimulatory blockade and improve allograft survival in T-cell-sensitized recipients (21). Another recent study reported that IFN-γ neutralization via blocking anti-IFN-γ antibody could prevent memory CD4^+^ T cells from providing CD40-independent help to B cells and thus improve allograft survival, which might be valuable for identifying transplant patients at risk for generation of *de novo* alloantibodies and for preventing alloantibody production in T-cell-sensitized recipients (22).

## Conclusion

Despite their role in protective immunity against invading pathogens, the presence of memory T cells also threatens transplant survival. The unique characteristics of memory T cells in protective immunity against recurrent pathogens also make them formidable barriers to desired tolerance and successful transplantation. Studies in experimental models have suggested that targeting the infiltration, proliferation, activation, and intrinsic apoptotic pathway of memory T cells may be promising therapeutic approaches. However, with any therapy designed to inhibit memory T-cell recall responses, there is an inherent risk of impairing pathogen-specific protective immune responses. Therefore, the benefits in terms of graft survival obtained by attenuating memory T-cell responses must be carefully weighed against the cost of compromised protective immunity, the risk of infection of the host, and the potential for the development of malignancy. Recent evidence, however, has shown fundamental differences in the recall responses to pathogens and allografts of memory T cells that may offer a therapeutic window in which “detrimental” graft-specific recall responses can be attenuated, while “beneficial” pathogen-derived responses remain unaffected.

## Conflict of Interest Statement

The authors declare that the research was conducted in the absence of any commercial or financial relationships that could be construed as a potential conflict of interest.
